# A Novel Temozolomide-Myricetin Drug-Drug Cocrystal: Preparation, Characterization, Property Evaluations

**DOI:** 10.3390/pharmaceutics17070906

**Published:** 2025-07-13

**Authors:** Hai-Xin Qin, Jie Wang, Jia-Hui Peng, Xia-Lin Dai, Cai-Wen Li, Tong-Bu Lu, Jia-Mei Chen

**Affiliations:** 1 School of Chemistry and Chemical Engineering, Tianjin University of Technology, Tianjin 300384, China; qinhaixin928@163.com (H.-X.Q.); pengjiahui@stud.tjut.edu.cn (J.-H.P.); daixialin@email.tjut.edu.cn (X.-L.D.); 2 Bionna (Beijing) Medicine Technology Co., Ltd., Beijing 102600, China; wangjie199707w@163.com; 3 Institute for New Energy Materials and Low Carbon Technologies, School of Materials Science and Engineering, Tianjin University of Technology, Tianjin 300384, China; lutongbu@tjut.edu.cn

**Keywords:** temozolomide, myricetin, drug-drug cocrystal, crystal structure, stability, tabletability, dissolution

## Abstract

**Objectives**: Drug-drug cocrystals with improved properties can be used to facilitate the development of synergistic therapeutic combinations. The goal of the present study is to obtain novel drug-drug cocrystals involving two anti-glioma agents, temozolomide (TMZ) and myricetin (MYR). **Methods**: The novel TMZ-MYR cocrystal was prepared via slurry and solvent evaporation techniques and characterized by X-ray diffraction, thermal analysis, infrared spectroscopy, and dynamic vapor sorption measurements. The stability, compaction, and dissolution properties were also evaluated. **Results**: Crystal structure analysis revealed that the cocrystal lattice contains two TMZ molecules, one MYR molecule, and four water molecules, which are linked by hydrogen bonding interactions to produce a three-dimensional network. The cocrystal hydrate exhibited favorable stability and tabletability compared to pure TMZ. A dissolution study showed that the maximum solubility of MYR in the cocrystal (176.4 μg/mL) was approximately 6.6 times higher than that of pure MYR·H_2_O (26.9 μg/mL), while the solubility of TMZ from the cocrystal (786.7 µg/mL) was remarkably lower than that of pure TMZ (7519.8 µg/mL). The solubility difference between MYR and TMZ was diminished from ~280-fold to ~4.5-fold. **Conclusions**: Overall, the TMZ-MYR cocrystal optimizes the stability and tabletability of TMZ and the dissolution behavior of both drugs, offering a promising approach for synergistic anti-glioma therapy with improved clinical potential.

## 1. Introduction

Pharmaceutical cocrystals assemble active pharmaceutical ingredients (APIs) with pharmaceutically acceptable cocrystal formers (CCFs) at a stoichiometric ratio through non-covalent interactions, forming a new type of homogeneous crystal with unique crystal structures and physicochemical properties [1]. The cocrystal formation can not only enhance the solubility and dissolution rate of insoluble drugs but also inhibit unstable chemical degradation pathways by altering the molecular stacking patterns, reduce the quality risk of drugs during storage or transportation, and improve the mechanical processing properties of powders to accelerate the development of formulations [2,3,4,5,6,7]. In addition, as new chemical entities, cocrystals can also provide new patent protection for pharmaceuticals and bring great economic benefits for companies [8]. Drug-drug cocrystals are a branch of pharmaceutical cocrystals, whose core feature is that the CCFs themselves are also APIs with therapeutic value [9,10]. As compared to physical mixtures or traditional compound preparations, drug-drug cocrystals can not only simplify administration plans and improve patients’ compliance but also effectively improve physicochemical properties and overcome the solubility differences and compatibility issues between different drugs [11].

**Figure 1:**
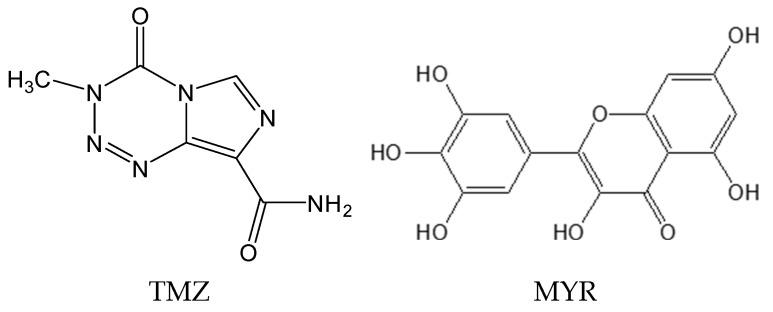
Chemical structures of TMZ and MYR.

Temozolomide (TMZ, Figure 1) is a first-line drug in the treatment of malignant gliomas with good anti-glioma activity and blood-brain barrier penetration ability [12]. TMZ is a white to slightly red solid powder with decomposition temperature of 212 °C. The pKa value of TMZ is 14.722 ± 0.20 (predicted). The aqueous solubility and log *p* value of TMZ are 5 mg/mL and −0.989 (predicted), respectively [13,14], so it is a BCS Class III drug. It is relatively stable under acidic conditions and undergoes hydrolysis under neutral or alkaline conditions [15]. TMZ has a rapid rate of elimination in vivo, a property that somewhat diminishes its therapeutic effect [16]. In addition, TMZ is prone to chemical degradation, resulting in a gradual reduction of its active ingredient and generation of some impurities, which in turn affects the safety and efficacy of the drug. Thus, TMZ faces significant challenges in areas such as production and storage [17]. Recently, two cocrystals of TMZ with baicalein and hesperetin have been reported and show improved properties as compared to the pristine drugs [18,19]. These findings inspired us to further explore the potential and possibility of constructing more TMZ cocrystals with flavonoids to offer more options for potential clinical application. Thus, a group of flavonoids with anti-glioma activity, including apigenin, daidzein, morin, myricetin, naringenin, etc. [20,21,22,23], were screened. Finally, we successfully obtained a novel drug-drug cocrystal of TMZ and myricetin (MYR, Figure 1).

MYR is a natural flavonoid and exhibits anti-glioma activity by blocking lamellipodia formation and suppressing focal adhesions [24]. Unlike baicalein and hesperetin, which induce glioma cell apoptosis via the reduction of the expression of HIF-1α, VEGF, and VEGFR2 and the activation of p38 MAPK [25,26], MYR employs a distinct mechanism, making it a promising candidate for combination therapy. MYR is a yellow needle-shaped crystalline powder with a melting point of 357 °C. The pKa value of MYR is 6.3 (experimental). The water solubility of MYR is 17 µg/mL, and its log *p* value is 1.655 (predicted) [14,27], thus it is classified as a BCS Class IV drug. MYR’s low bioavailability due to its poor solubility limits its clinical utility [28]. To address these challenges, we focused on developing a novel TMZ-MYR drug-drug cocrystal. The objective was to optimize the properties of both drugs for synergistic anti-glioma treatment. This study reports the preparation, characterization, and property evaluations of the TMZ-MYR cocrystal. The cocrystal’s crystal structure, moisture sorption behavior, stability, compaction property, and dissolution profiles were systematically investigated to demonstrate its potential as an improved therapeutic formulation for glioma.

## 2. Materials and Methods

**Materials.** Temozolomide (TMZ, 98% purity) and myricetin monohydrate (MYR∙H_2_O, 98% purity) were obtained from Shanghai Shengde Pharmaceutical Science and Technology Co., Ltd. (Shanghai, China). Butanone (analytical grade), acetic acid (analytical grade), sodium chloride (NaCl, analytical grade), and potassium bromide (KBr, spectroscopic grade) were purchased from Fuchen Chemical Reagent Co., Ltd. (Tianjin, China). Hydrochloric acid (analytical grade) was supplied by Sinopharm Chemical Reagent Co., Ltd. (Shanghai, China). Tween 80 (pharmaceutical grade) was acquired from Shanghai Macklin Biochemical Co., Ltd. (Shanghai, China). Anhydrous methanol (chromatographic purity), used as the eluent in HPLC analyses, was purchased from Tianjin Saifurui Technology Co., Ltd. (Tianjin, China). All chemicals and solvents were used as received without further purification.

**Preparation of Cocrystal, 2TMZ/MYR·4H_2_O (2:1:4).** The cocrystal was prepared by two methods as follows: (I) TMZ (0.4 mmol, 77.6 mg) and MYR∙H_2_O (0.2 mmol, 63.6 mg) were accurately weighed into a 10 mL Eppendorf tube and 1 mL of water was added. The reaction was carried out on a magnetic stirrer (RCT D S025, IKA, Staufen, Germany) at a stirring rate of 500 rpm for 12 h. At the end of the reaction, the suspension was filtered by a filter (SHB-Ⅲ, Tianjin Kenuo Instrument and Equipment Co., Ltd., Tianjin, China) and the filter cake was allowed to dry under vacuum for 48 h. The cocrystal powder (130.0 mg) was obtained in 92.07% yield. (II) Excess of cocrystal powder was placed in 2 mL of butanone and processed using an ultrasonic instrument (KS-040AL, Shenzhen Jiekang Cleaning Electrical Appliance Co., Ltd., Shenzhen, China) at room temperature for 20 min to fully mix and dissolve. The resulting solution was filtered through a 0.22 μm organic nylon filter, and the filtrate was transferred to a clean high-borosilicate glass beaker, which was then sealed with Parafilm. After one week, needle-like crystals were obtained.

**Single-Crystal X-ray Diffraction.** Single crystals of **2TMZ/MYR·4H_2_O** were mounted on an Agilent Technologies Gemini A Ultra system and irradiated with monochromated Cu Kα radiation (λ = 1.54178 Å) at 293(2) K. Cell refinement and data reduction were conducted with the CrysAlisPro software ver.1.171.44.91a [29]. The crystal structure was solved by the direct method using SHELX-97 [30] and refined by full-matrix least squares on *F*^2^ with Olex2 ver. 1.5. Non-hydrogen atoms were refined with anisotropic displacement parameters. Hydrogen atoms were placed in geometrically idealized positions and refined using a riding model.

**Powder X-ray Diffraction (PXRD).** PXRD measurement was conducted on a Rigaku MiniFlex 600 X-ray diffractometer (Rigaku, Akishima-shi, Japan) with Cu Kα radiation (λ = 1.541862 Å) as a light source. The voltage and current were 40 kV and 15 mA. The step size and scanning speed were set as 0.01°/step and 0.1 s/step, and the scanning range was set as 5–40°. The data collection and processing were conducted using SmartLab Studio II software ver. 4.3.287.0.

**Thermogravimetric (TG) Analysis.** TG analysis was conducted using a Netzsch TG 209 F3 analyzer (Netzsch, Selb, Germany) from 40 to 500 °C at 10 °C/min. Both the purge gas and protective gas were nitrogen, with flow rates of 50 mL/min and 20 mL/min, respectively. Samples (5–10 mg) were placed on alumina crucibles, and data collection and processing were conducted using NETZSCH Proteus thermal analysis software ver. 7.1.0.

**Differential Scanning Calorimetry (DSC).** DSC measurements were performed using a Netzsch DSC 200 F3 instrument (Netzsch, Selb, Germany) from 30 °C to the decomposition temperature of each sample (206 °C for TMZ, 350 °C for MYR∙H_2_O, 300 °C for the cocrystal) at 10 °C/min in a nitrogen atmosphere. Samples (5–10 mg) were sealed in aluminum pans, and data collection and processing were conducted using NETZSCH Proteus thermal analysis software ver. 7.1.0.

**Fourier Transform Infrared (FTIR) Spectroscopy.** FTIR spectra were acquired on a BRUKER VERTEX 70 spectrometer (Bruker, Karlsruhe, Germany) using the KBr pellet method. The mass ratio of powder sample to dried KBr is 1:100. For sample preparation, the sample and KBr were ground uniformly in a mortar, then pressed into a transparent pellet using a tablet press under a pressure of 1.0 t for 2 min, which was subsequently used for analysis. Spectra were scanned over the range of 4000 to 500 cm^−1^ with 64 scans and a resolution of 0.2 cm^−1^. Data collection and processing were performed using Spectrum software ver. 10.4.4.449.

**Dynamic Vapor Sorption (DVS) Study.** A DVS study was performed on a DVS intrinsic instrument (Surface Measurement Systems, London, UK) at 25 ± 0.1 °C. Sieved samples (75–150 μm) were exposed to a humidity gradient from 0% to 95% RH and back to 0%, in 10% RH steps. Weight changes were recorded to evaluate moisture sorption behavior. The instrument was controlled using DVS Intrinsic Control Software ver. 1.0.3.1, and data processing was conducted using the Isotherm (ISO) analysis suite.

**Accelerated Stability Test.** The samples of the cocrystal and TMZ were weighed into open vials and then stored in a stability chamber (Memmert, Schwabach, Germany) at 40 °C. A relative humidity of 75% was maintained using a sealed desiccator containing a saturated NaCl solution. Samples were analyzed at 0, 1, 2, and 3 months using PXRD and HPLC to monitor phase purity and TMZ content. Visual inspection was performed to assess physical changes.

**Powder Compaction Experiment.** Powder compaction properties of **2TMZ/MYR·4H_2_O**, pure TMZ, and MYR·H_2_O were evaluated using a reported method [19]. Powder compaction was performed using a hydraulic press (Specac GS01190, New York, NY, USA) at pressures from 50 to 300 MPa with a 5 mm die. The tablet breaking force was tested by an intelligent tablet hardness analyzer (YD-20KZ, TDTF, Tianjin, China). Tablet tensile strength (σ) was calculated using Equation (1),

(1) $$\sigma =\frac{2F}{\pi Dt}$$

where *F* is the breaking force, *D* is tablet diameter, and *t* is the thickness. Tensile strength measurements were repeated three times to ensure reproducibility. PXRD tests were conducted to detect possible phase changes for the samples compressed under 300 MPa.

**Powder Dissolution Experiment.** Powder dissolution experiments of **2TMZ/MYR·4H_2_O**, pure TMZ, and MYR·H_2_O were carried out according to a reported method [19]. The drug dosage used in the dissolution experiments was equivalent to 700 mg TMZ and 574 mg MYR. The dissolution experiments were conducted in a paddle apparatus with 45 mL of pH 1.2 HCl containing 0.1% Tween 80 at 37 °C and 100 rpm. Samples were withdrawn at scheduled time points, filtered, and analyzed by HPLC. Powder dissolution experiments were repeated three times to evaluate standard deviations. After the dissolution experiments, the remaining solids were collected for PXRD analysis.

**High Performance Liquid Chromatography (HPLC) Analysis.** HPLC analysis was performed following a modified protocol based on previously reported methods [19]. A Shimadzu LC-2030C 3D Plus HPLC system equipped with a C18 column (Inertsil ODS-3, 5 μm × 4.6 mm × 150 mm column) was utilized. The detection wavelengths were set at 329 nm for TMZ and 375 nm for MYR. The column temperature was maintained at 35 °C throughout the analysis. The mobile phase consisted of a mixture of methanol and an acetic acid solution (adjusted to pH 2.8). Gradient elution was employed with a constant flow rate of 1 mL/min, following the sequence: 10% (*v/v*) methanol for 7 min, linear increase to 60% (*v/v*) methanol in 1 min, isocratic elution at 60% (*v/v*) methanol for 8 min, return to 10% (*v/v*) methanol over 1.5 min, re-equilibration at 10% (*v/v*) methanol for 2.5 min. This method enabled the simultaneous quantification of TMZ and MYR in the cocrystal samples, ensuring accurate determination of their concentrations and purity. Data collection and processing were performed using LabSolutions software ver. 5.96.

## 3. Results and Discussion

**Cocrystal Preparation.** The phase-pure bulk sample of the cocrystal hydrate, **2TMZ/MYR·4H_2_O**, was prepared by a slurry technique. This cocrystal has a unique 2:1 stoichiometric ratio of TMZ and MYR, differing from the 1:1 ratio of previously reported TMZ-flavonoid cocrystals [18,19]. This distinction provides an additional clinical option for personalized glioma therapy. The high-quality single crystals were obtained via recrystallization in butanone at room temperature, which were essential for subsequent crystal structure analysis.

**Figure 2:**
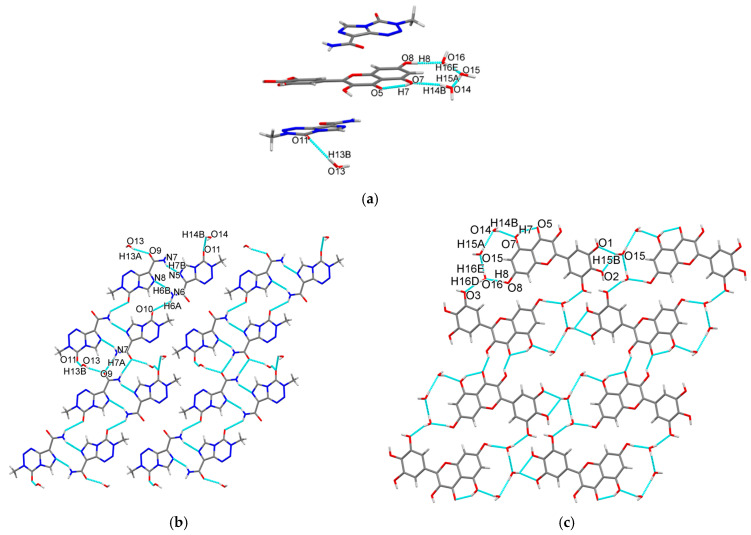

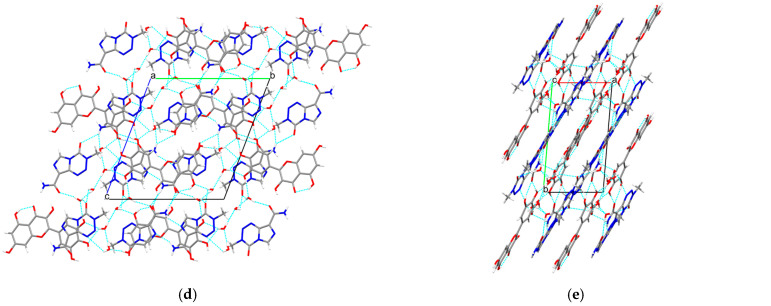
(**a**) Asymmetric unit, (**b**) TMZ sheet, (**c**) MYR sheet, (**d**) top and (**e**) side view of 3D structure of **2TMZ/MYR·4H_2_O**.

**Crystal Structure Analysis.** The crystallographic data and refinement parameters of the cocrystal are summarized in Table 1. Selected hydrogen bonding distances and angles are listed in Table S1. The cocrystal crystallizes in the triclinic space group *P*-1, showing lattice constants *a* = 7.4917(5) Å, *b* = 14.4652(10) Å, *c* = 15.9455(10) Å, *α* = 110.295(6)°, *β* = 100.265(5)°, *γ* = 90.811(5)° (Table 1). The asymmetric unit contains two TMZ molecules, one MYR molecule, and four water molecules. Hydrogen bonds link one TMZ molecule to one water molecule and MYR to three water molecules (Figure 2a). TMZ and water molecules form a two-dimensional (2D) planar structure via hydrogen bonds (Figure 2b), and MYR also forms another type of 2D hydrogen-bonded plane with water molecules (Figure 2c). These two types of 2D planes then stack in an alternating arrangement via hydrogen bonds and π–π interactions, ultimately constructing a three-dimensional (3D) supramolecular structure (Figure 2d,e).

Crystal engineering analysis reveals that the parent drug TMZ (reference code DIPGIS10) and MYR·H_2_O (reference code NIKLAX) both crystallize in the monoclinic *P*2_1_/c space group [31,32]. TMZ forms dimers through amide-amide R_2_^2^(8) double hydrogen bonds (Figure S1a), and four dimers are connected by weak hydrogen bonds to form cyclic nodes (Figure S1b). These nodes periodically assemble into a reticular interwoven framework structure (Figure S1c). Meanwhile, intramolecular hydrogen bonds exist between the carbonyl and adjacent hydroxyl group of the benzene ring in MYR·H_2_O, and MYR molecules form wavy hydrogen-bonded structures through other hydroxyls on two benzene rings (Figure S2a). These wavy structures stack into a 3D structure via π–π interactions and hydrogen bonds of water molecules (Figure S2b). Cocrystallization retains the amide-amide R_2_^2^(8) dimer of TMZ and the intramolecular hydrogen bond of MYR, while rebalancing other hydrogen bond donors/acceptors; the crystal packing mode changes from the reticular interwoven framework of TMZ and the wavy stacking of MYR to an alternating layered architecture of TMZ-MYR, thereby affecting the stability, tabletability, and dissolution behavior of the cocrystal.

**Figure 3:**
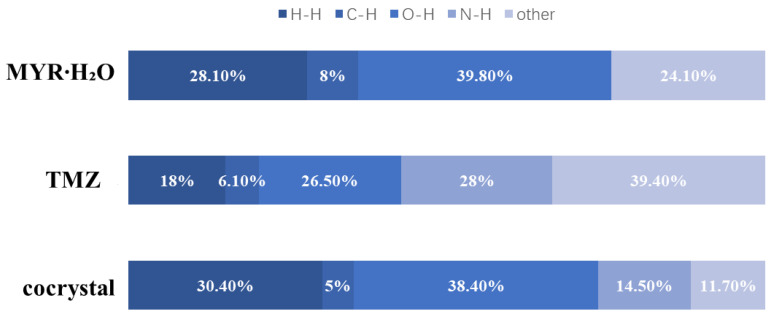
Percentage contributions of intermolecular interactions for TMZ, MYR·H_2_O, and **2TMZ/MYR·4H_2_O**.

**Hirshfeld Surface Analysis.** Hirshfeld surface analysis is a tool for quantifying and visualizing intermolecular contacts [33]. Based on the single-crystal structures of the cocrystal and its component crystals, Hirshfeld surface analysis was performed. Two-dimensional fingerprint plots are shown in Figure S3, and the relative contributions of various intermolecular contacts are illustrated in Figure 3. For **2TMZ/MYR·4H_2_O** and its component crystals, the main contributions come from H-H, C-H, and O-H contacts, corresponding to van der Waals forces, C-H···π interactions, and hydrogen bonds, respectively. Additionally, pure TMZ and the cocrystal include N-H contacts (Figure 3). Cocrystallization significantly alters the intermolecular interaction patterns. Compared with pure TMZ, the contributions of O-H (26.5%→38.4%) and H-H (18%→30.40%) increase in the cocrystal, reflecting enhanced hydrogen bonding between TMZ and hydrophobic MYR as well as stronger van der Waals forces, which may reduce the solubility of TMZ. For MYR, the cocrystal shows increased contribution N-H (0%→14.5%), while the contribution of C-H (8%→5%) decreases, indicating enhanced hydrogen bonding with hydrophilic TMZ and weakened C-H···π interactions, which may improve the dissolution performance of MYR.

**Figure 4:**
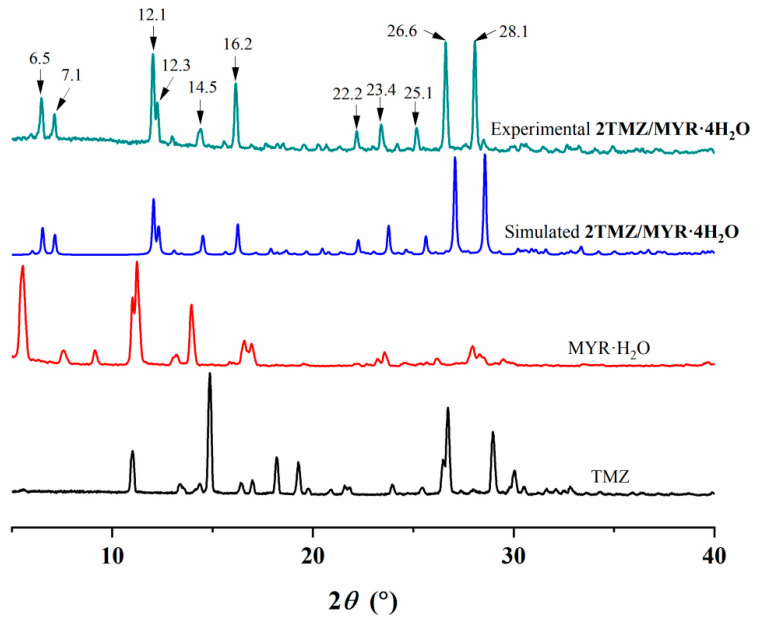
PXRD patterns of **2TMZ/MYR·4H_2_O**, MYR·H_2_O, and TMZ.

**PXRD Analysis.** The experimental PXRD pattern of the cocrystal matched the simulated pattern derived from single-crystal data, confirming its crystalline phase purity. Distinct peaks at 2*θ* values of 6.5°, 7.1°, 12.1°, 12.3°, 14.5°, 16.2°, 22.2°, 23.4°, 25.1°, 26.6°, and 28.1° were observed, which were absent in the patterns of pure TMZ and MYR·H_2_O (Figure 4). This result indicates the formation of a new crystalline phase, distinguishing the cocrystal from its parent materials.

**Figure 5:**
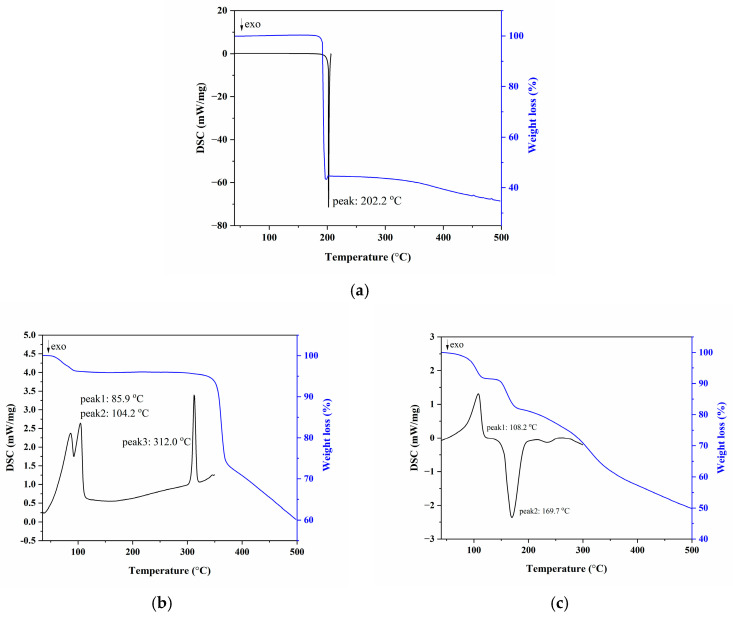
TG and DSC thermograms of (**a**) TMZ, (**b**) MYR·H_2_O, and (**c**) **2TMZ/MYR·4H_2_O**.

**Thermal Analysis.** DSC and TG measurements revealed the thermal behavior of the cocrystal (Figure 5). The TG curve showed a 9.3% weight loss below 120 °C, corresponding to the loss of four water molecules (theoretical 9.25%). The DSC curve exhibited an endothermic peak at 108.2 °C due to dehydration, followed by an exothermic peak at 169.7 °C from TMZ decomposition. In comparison, pure TMZ decomposed with a sharp exothermic peak at 202.2 °C, while MYR·H_2_O showed dehydration, dehydration-induced phase transition, and melting point with endothermic peaks at 85.9 °C, 104.2 °C, and 312.0 °C, respectively. The thermal analysis suggests that cocrystallization modifies the thermal properties of both drugs, potentially improving their stability during storage and processing.

**Figure 6:**
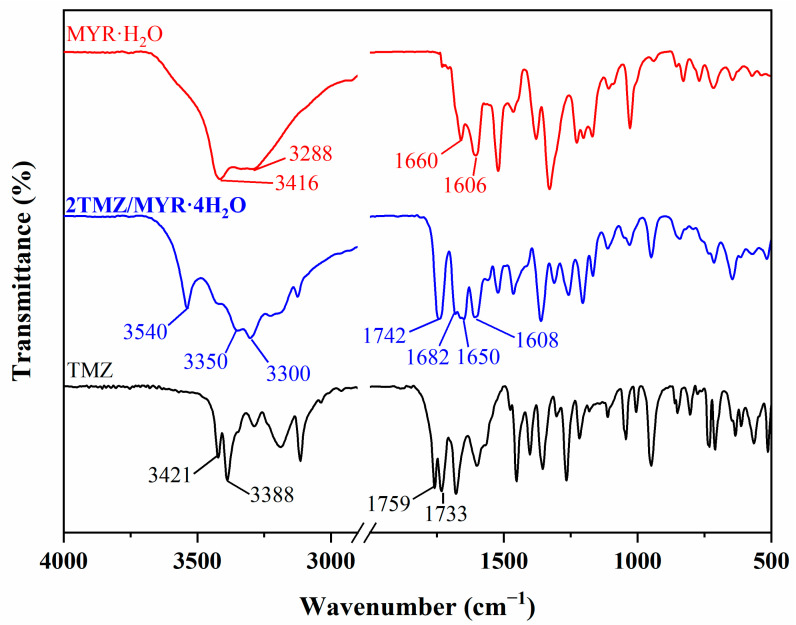
FTIR spectra of TMZ, MYR·H_2_O, and **2TMZ/MYR·4H_2_O**.

**FTIR Analysis.** FTIR spectra can be used to confirm the formation of hydrogen bonds in the cocrystal. The FTIR spectra of TMZ, MYR·H_2_O, and **2TMZ/MYR·4H_2_O** are provided in Figure 6. For pure TMZ, the characteristic peaks presented at 3421 and 3388 cm^−1^ are attributed to the stretching vibrations of the N−H bond, and the C=O stretching vibrations appeared at 1759 and 1733 cm^−1^ [34]. MYR·H_2_O showed characteristic peaks at 1606, 1660 cm^−1^ and 3288, 3416 cm^−1^ which were stretching vibrations of carbonyl and hydroxyl groups, respectively. After the formation of the cocrystal, the C=O stretching vibrations were observed at 1608, 1650 and 1682, 1742 cm^−1^ and the N−H and O−H stretching were shifted to 3300, 3350, and 3540 cm^−1^, respectively. Similar to the reported TMZ-hesperetin cocrystal [19], the FTIR bands corresponding to the amide groups of TMZ and the carbonyl groups of flavonoids underwent red shift in hydrogen bonding modes with the formation of cocrystal. However, there are differences in the changes of the characteristic peaks of the hydroxyl groups of flavonoids after cocrystal formation. The hydroxyl peak of hesperetin exhibited a red shift, while that of myricetin underwent a blue shift due to the interactions with water molecules in the cocrystal lattice.

**Figure 7:**
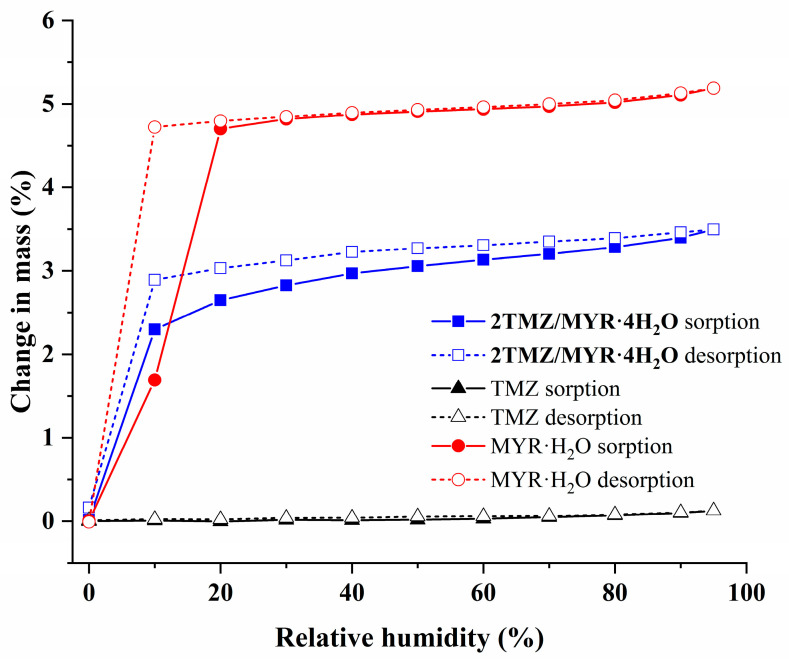
Water sorption/desorption isotherms of TMZ, MYR·H_2_O, and **2TMZ/MYR·4H_2_O** at 25 °C.

**DVS Study.** A DVS study of TMZ, MYR·H_2_O, and **2TMZ/MYR·4H_2_O** was performed and the resulting vapor sorption/desorption isotherms are shown in Figure 7. PXRD tests were used to detect possible phase changes (Figure S4). The results showed that the cocrystal and MYR·H_2_O exhibited humidity-induced phase transitions, while TMZ remained stable across all relative humidity (RH) ranges. At 0% RH, both the cocrystal and MYR·H_2_O lost one water molecule, forming TMZ-MYR·3H_2_O and anhydrous MYR, respectively. Upon increasing RH to 10% and 20%, they absorbed one water molecule back and reverted to their original hydrated forms, demonstrating reversible water adsorption/desorption. Among the four water molecules in the cocrystal, one (O15 in Figure 2a) only forms hydrogen bonds with the other two water molecules and lacks strong hydrogen bonding interactions with MYR or TMZ. This weaker interaction profile makes it more prone to desorption. The cocrystal is stable between 10% and 95% RH, making it suitable for pharmaceutical formulations.

**Figure 8:**
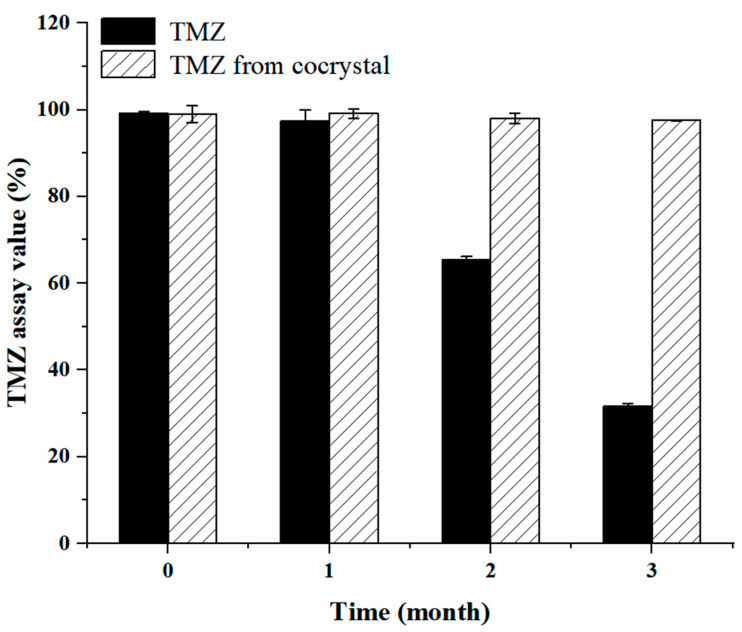
Changes in TMZ assay values of pure TMZ and the cocrystal during storage under 40 °C/ 75% RH (*n* = 3).

**Accelerated Stability Study.** Considering that TMZ is subject to stability issues, an accelerated stability study was conducted to evaluate the physical and chemical stability of the cocrystal and TMZ under stressed conditions (40 °C/75% RH). The cocrystal maintained its yellow crystalline appearance for 3 months, while pure TMZ showed signs of discoloration within 1 month (Figure S5). The cocrystal’s PXRD pattern remained unchanged throughout the study. In contrast, pure TMZ exhibited new degradation peaks at 2*θ* values of 11.6°, 12.9°, and 27.3° by the 2nd month, indicating crystalline phase changes (Figure S6). Additionally, the residual TMZ content in the cocrystal was 97.59 ± 0.14% after 3 months, significantly higher than that of pure TMZ (31.64 ± 0.64%) (Figure 8). These results confirm that the cocrystal retained its structural integrity under accelerated conditions, whereas TMZ underwent degradation as previously reported [35]. Compared to previous study on TMZ-flavonoid cocrystals, **2TMZ/MYR·4H_2_O** demonstrates comparable or superior stability. For example, the TMZ-hesperetin cocrystal retained 95.3% of TMZ after 3 months under similar conditions [19], whereas the present cocrystal maintains 97.59% TMZ content, highlighting the unique stabilizing effect of MYR. The cocrystal’s improved stability can be attributed to its 3D hydrogen-bonded network, which restricts molecular mobility and shields labile functional groups by disrupting the self-assembled reticular framework of TMZ (Figure S1). This structural arrangement effectively suppresses TMZ decomposition.

**Compaction Property.** Compaction properties of the cocrystal, pure TMZ, and MYR·H_2_O were evaluated by powder compaction experiments. Figure S7 shows typical tablets of each sample compressed at 300 MPa. Under a compression pressure of up to 300 MPa, TMZ powder cannot form intact tablets, showing tablet lamination and capping. In contrast, MYR·H_2_O and **2TMZ/MYR·4H_2_O** powders can be made into intact tablets throughout the entire compaction pressure range, demonstrating significantly improved compression performance. Additionally, these tablets remained intact after 24 h of relaxation, with no signs of lamination or capping.

**Figure 9:**
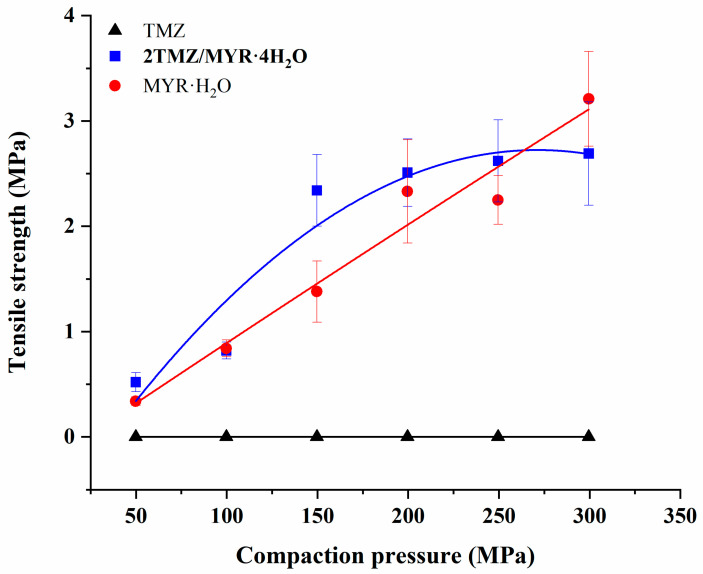
Tabletability of TMZ, MYR∙H_2_O, and **2TMZ/MYR·4H_2_O** (*n* = 3).

The tabletability profiles of the three powdered samples from 50 MPa to 300 MPa are shown in Figure 9. For the cocrystal and MYR·H_2_O, tensile strength increased with compaction pressure, reaching the pharmaceutical tablet threshold (≥2 MPa) above 200 MPa [36]. They exhibited superior tabletability compared to pure TMZ. The compaction performance of the materials was correlated with their crystal structures [37,38]. The reticular interwoven framework of TMZ (Figure S1c) limits plastic deformation, leading to low tensile strength and poor tablet integrity. The cocrystal and MYR·H_2_O form 2D layered and wavy structures (Figure 2d,e and Figure S2c), with the existence of crystalline water molecules in their structures, which enhance plastic deformation and improve tabletability. Compared to other TMZ-flavonoid cocrystals, **2TMZ/MYR·4H_2_O** shows better tabletability [19]. PXRD analysis of the compacted samples revealed no phase transitions of the cocrystal or MYR·H_2_O under 300 MPa (Figure S8), which is crucial for maintaining drug efficacy in tablet formulations.

**Figure 10:**
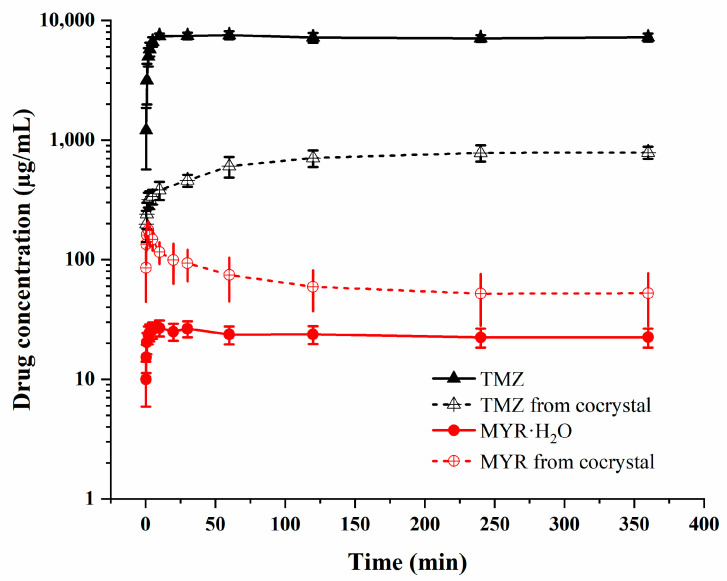
Powder dissolution profiles of TMZ, MYR∙H_2_O, and the cocrystal (*n* = 3).

**Dissolution Study.** A dissolution study was conducted to evaluate the solubility and release profiles of TMZ and MYR from the cocrystal, compared to their pure forms. The cocrystal showed an improved MYR dissolution rate while TMZ release from the cocrystal was delayed compared to pure TMZ (Figure 10). The maximum apparent solubility (*S*_max_) of MYR in the cocrystal was 176.4 μg/mL, significantly higher than that of pure MYR·H_2_O (26.9 μg/mL)—an approximately 6.6-fold increase. The cocrystal’s hydrogen-bonded 3D network weakens the intermolecular interactions of MYR itself, facilitating solvent penetration and dissolution. Like other TMZ-flavonoid cocrystals, **2TMZ/MYR·4H_2_O** also shows superior MYR solubility enhancement [18,19]. This enhancement may address MYR’s low bioavailability, which is typically limited by its poor water solubility. TMZ solubility from the cocrystal (786.7 μg/mL) was remarkably lower than that of pure TMZ (7519.8 μg/mL). The reduction in TMZ solubility can be attributed to the formation of the cocrystal between TMZ and hydrophobic MYR, which reduces TMZ’s affinity for aqueous media, resulting in a slower yet more controlled release profile. The solubility gap between TMZ and MYR was drastically reduced from ~280-fold (in pure forms) to ~4.5-fold in the cocrystal. This balance in release profiles is critical for synergistic anti-glioma therapy, as it ensures concurrent availability of both drugs. In contrast, physical mixtures exhibit mismatched release kinetics, limiting their clinical efficacy. Following the powder dissolution experiment, the crystal phases of the residual solids were analyzed. TMZ was found to transform into TMZ·H_2_O, whereas the crystal phase of MYR·H_2_O remained unchanged. In the **2TMZ/MYR·4H_2_O** cocrystal, the diffraction peak corresponding to MYR·H_2_O emerged, which can be ascribed to the dissociation of the two components in the dissolution medium. Given the poor solubility of MYR·H_2_O and the good solubility of TMZ, excess MYR·H_2_O precipitated, leading to the appearance of its peaks in the sample (Figure S9).

## 4. Conclusions

In this study, a novel drug-drug cocrystal hydrate of TMZ and MYR, denoted as **2TMZ/MYR·4H_2_O**, was successfully prepared via slurry and solvent evaporation techniques. The cocrystal was fully characterized using XRD, TG, DSC, FTIR, and DVS measurements. Crystal structure analysis revealed that the cocrystal lattice contained two TMZ molecules, one MYR molecule, and four water molecules, which were linked by hydrogen bonding interactions to produce a 3D network. Property evaluations demonstrated significant improvements in the stability and tabletability of TMZ, as well as the enhancement of solubility of MYR, and the delayed dissolution behavior of TMZ. In summary, the cocrystal represents a promising strategy for synergistic anti-glioma therapy, combining the therapeutic effects of TMZ and MYR while overcoming their individual limitations.

## Figures and Tables

**Table 1:** Crystallographic data and refinement parameters of **2TMZ/MYR·4H_2_O**.

Compound	2TMZ/MYR·4H_2_O
chemical formula	C_27_H_30_N_12_O_16_
formula wt	778.63
temperature (K)	293(2)
crystal size (mm^3^)	0.10 × 0.05 × 0.08
crystal system	triclinic
space group	*P*-1
*a* (Å)	7.4917(5)
*b* (Å)	14.4652(10)
*c* (Å)	15.9455(10)
*α* (deg)	110.295(6)
*β* (deg)	100.265(5)
*γ* (deg)	90.811(5)
volume (Å^3^)	1589.37(19)
*Z*	2
calculated density (g/cm^3^)	1.627
*θ* range for data collection	3.013–63.683
F (000)	808
index ranges	−8 ≤ h ≤ 8
	−16 ≤ k ≤ 16
	−17 ≤ l ≤ 18
no. of reflns	5231
no. of unique reflns	4372
no. of params	524
*R*_all_, *R*_obs_ *^a^*	0.0698, 0.0602
*wR*_2,all_, *wR*_2,obs_ *^b^*	0.1581, 0.1484
GOF	1.058
CCDC No.	2456974

*^a^ R*_1_ = Σ||*F_o_*| − |*F_c_*||/Σ|*F_o_*|. *^b^ wR*_2_ = [Σ[*w*(*F_o_^2^* − *F_c_^2^*)^2^]/Σ*w*(*F_o_^2^*)^2^]^1/2^, *w* = 1/[σ^2^ (*F_o_*)^2^ + (*aP*)^2^ + *bP*], where *P* = [(*F_o_*^2^) + 2*F*_c_^2^]/3.

## Data Availability

The results obtained for all experiments performed are shown in the manuscript and Supplementary Materials, the raw data will be provided upon request.

## Supplementary Materials

The following supporting information can be downloaded at: https://www.mdpi.com/article/10.3390/pharmaceutics17070906/s1, Figure S1: (a) Dimer, (b) ring-shaped node, and (c) reticular interlaced structure of TMZ crystal; Figure S2: (a) Top and (b) side view of 2D wavy structure, and (c) 3D structure of MYR∙H_2_O; Figure S3: Hirshfeld Surface 2D fingerprint plots of (a) TMZ, (b) MYR∙H_2_O, and (c) **2TMZ/MYR·4H_2_O**; Figure S4: PXRD patterns of TMZ, MYR∙H_2_O, **2TMZ/MYR·4H_2_O** before and after DVS measurements; Figure S5: Photographs of (a) TMZ and (b) **2TMZ/MYR·4H_2_O** under 40 °C/75% RH at 0, 1, 2, and 3 months. Figure S6: PXRD patterns of (a) TMZ and (b) **2TMZ/MYR·4H_2_O** under 40 °C/75% RH; Figure S7: Photographs of (a) TMZ, (b) MYR∙H_2_O, and (c) **2TMZ/MYR·4H_2_O** tablets under compaction pressure of 300 MPa; Figure S8: PXRD patterns of MYR∙H_2_O and **2TMZ/MYR·4H_2_O** before and after compaction experiments at 300 MPa; Figure S9: PXRD patterns of TMZ, MYR∙H_2_O, **2TMZ/MYR·4H_2_O** before and after dissolution experiments; Table S1: The hydrogen bonding distances and angles of **2TMZ/MYR·4H_2_O**.
