# Supplementary material for: A Novel Temozolomide-Myricetin Drug-Drug Cocrystal: Preparation, Characterization, Property Evaluations
Source: Pharmaceutics. 2025 Jul 13;17(7):906. doi: 10.3390/pharmaceutics17070906 (PMC12299715; doi:10.3390/pharmaceutics17070906)
Supplement: Supplementary file 1 [file pharmaceutics-17-00906-s001.zip › Revised Supplementary Materials.docx]

Supplementary Materials for

**A Novel Temozolomide-Myricetin Drug-Drug Cocrystal: Preparation, Characterization, Property Evaluations**

**Hai-Xin Qin, Jie Wang, Jia-Hui Peng, Xia-Lin Dai, Cai-Wen Li, Tong-Bu Lu, Jia-Mei Chen**


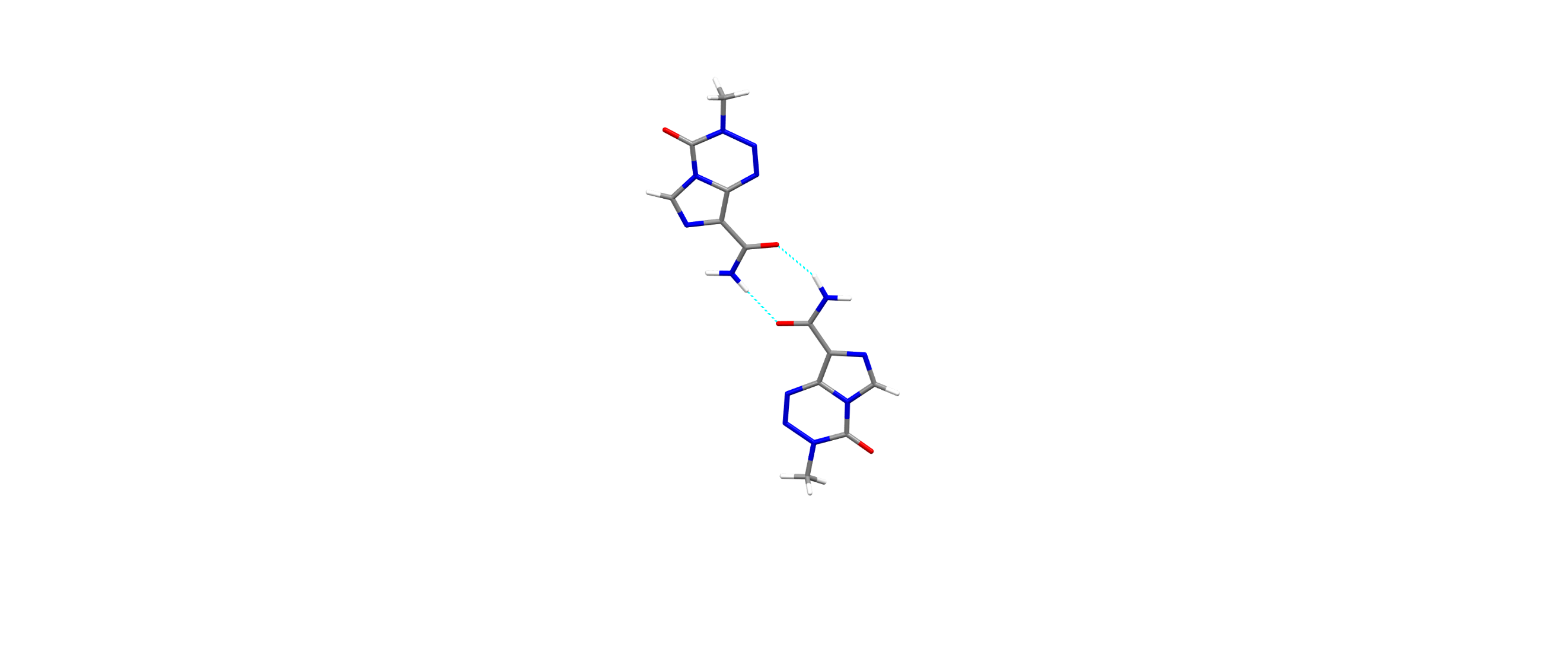

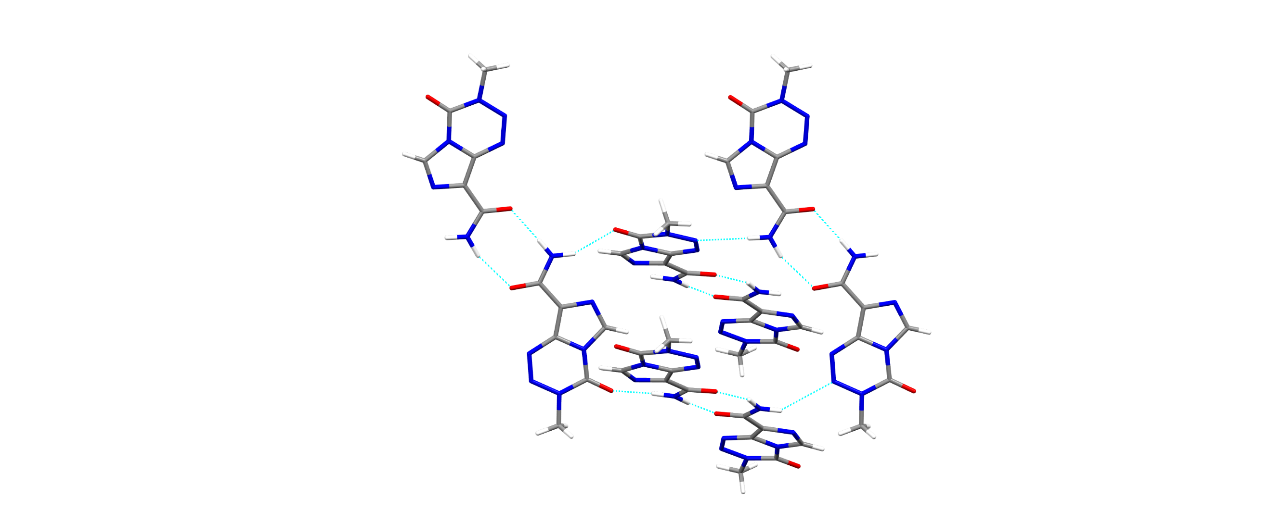

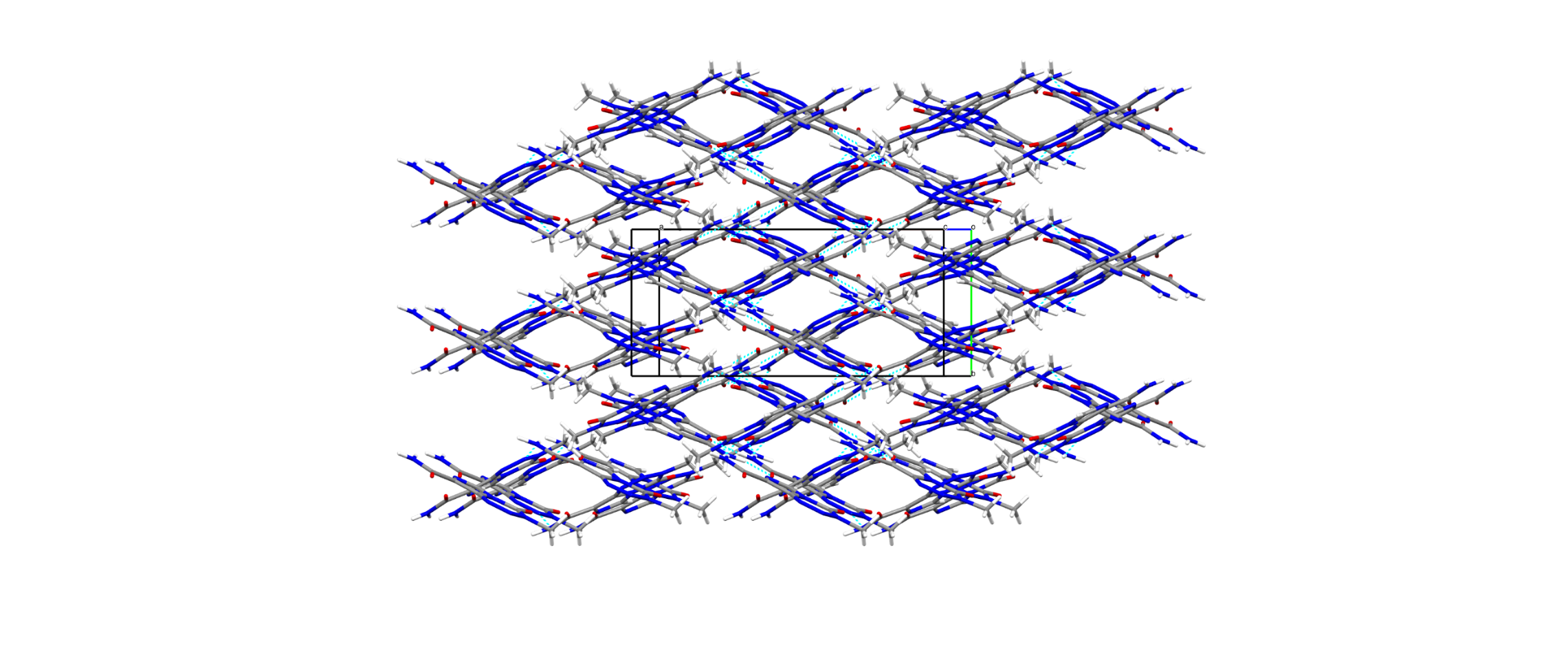


**(c)**

**(b)**

**(a)**

**Figure S1.** (a) Dimer, (b) ring-shaped node, and (c) reticular interlaced structure of TMZ crystal.

**
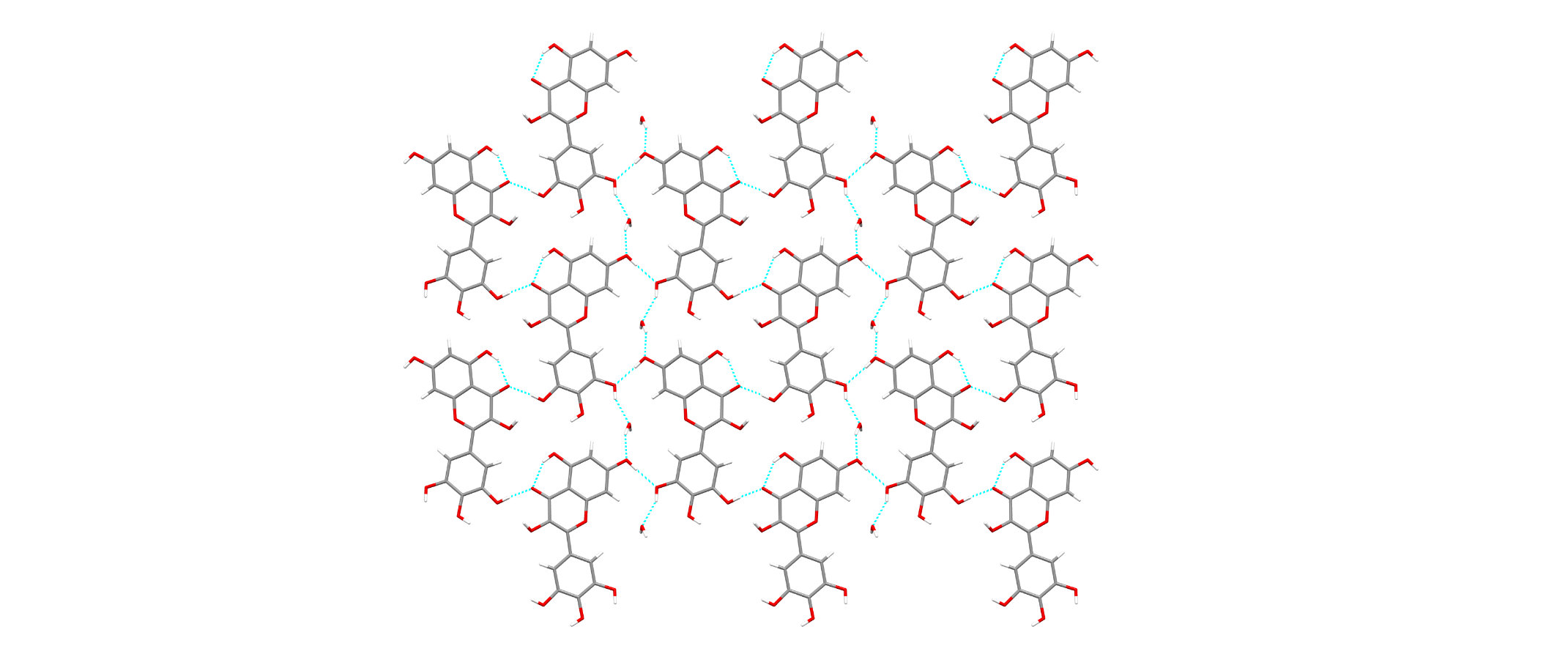

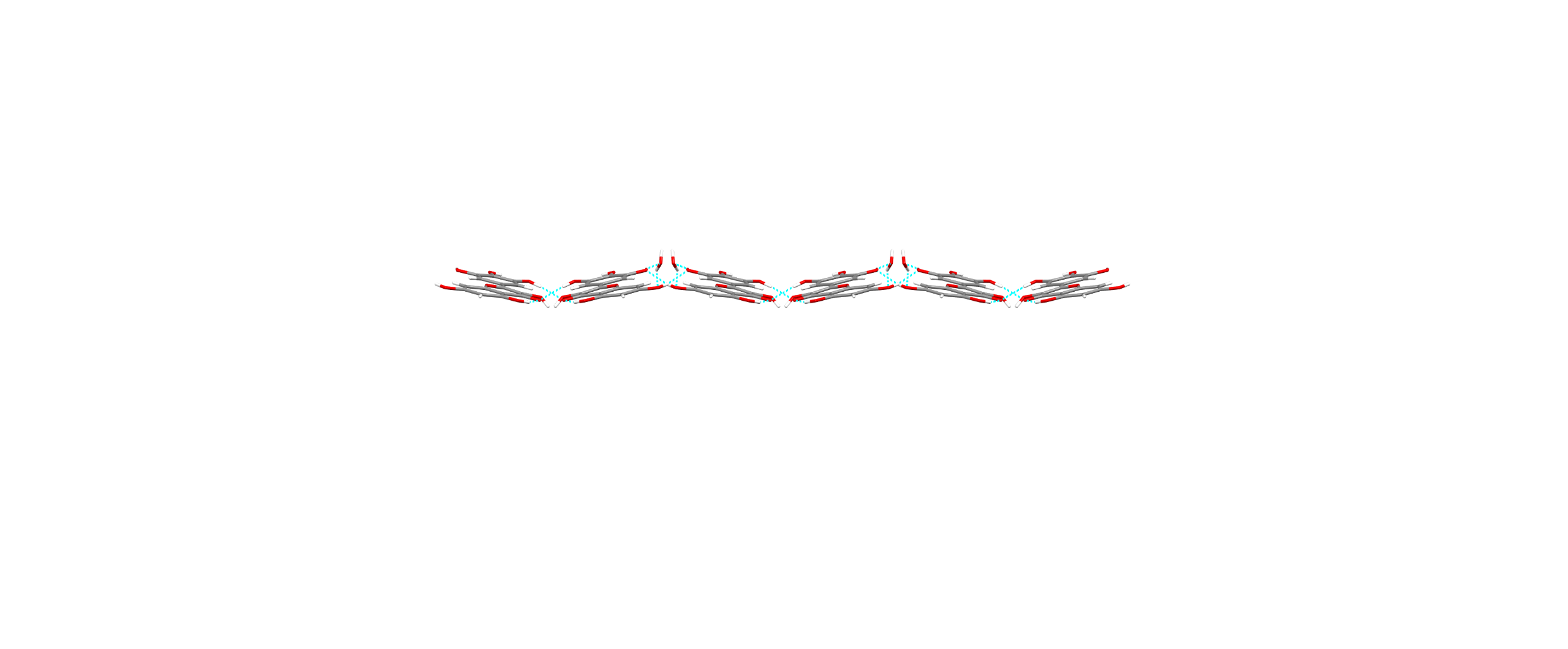

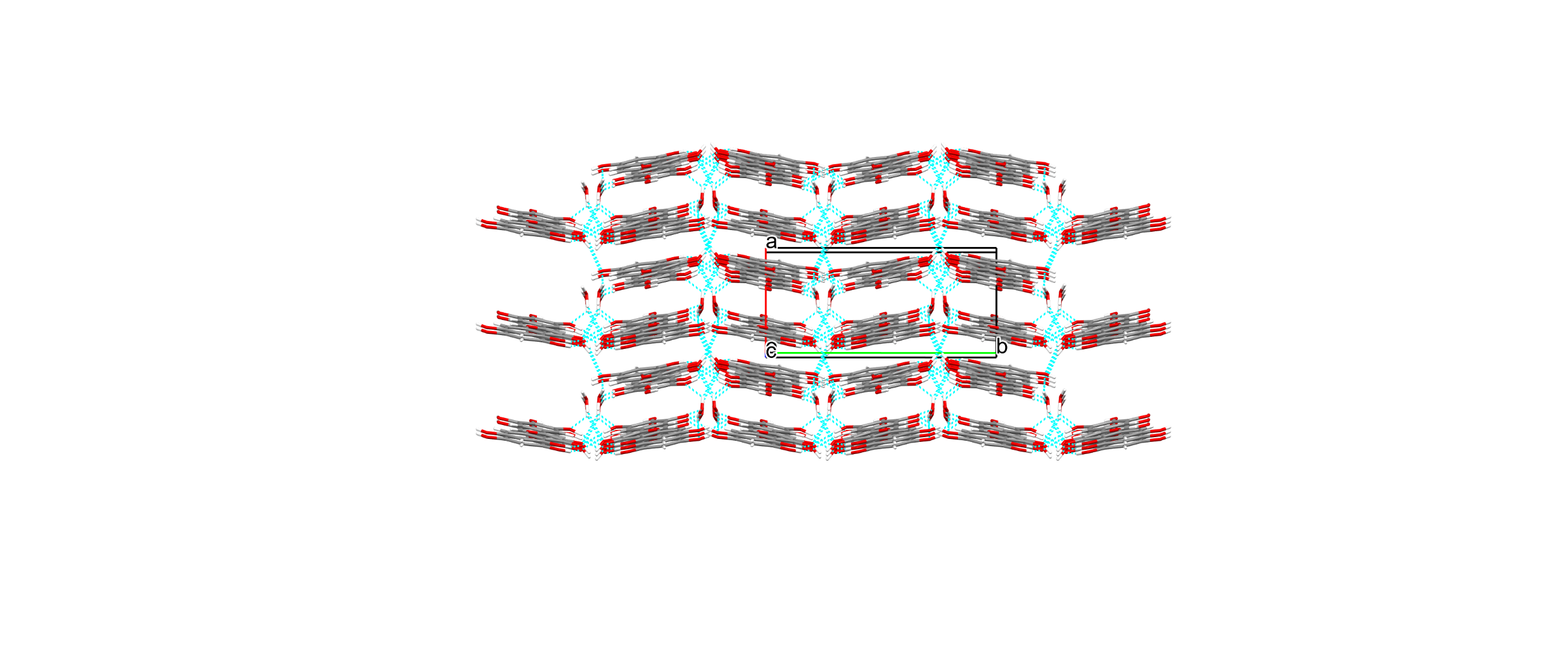
**

**(b)**

**(c)**

**(a)**

**Figure S2.** (a) Top and (b) side view of 2D wavy structure, and (c) 3D structure of MYR∙H₂O.


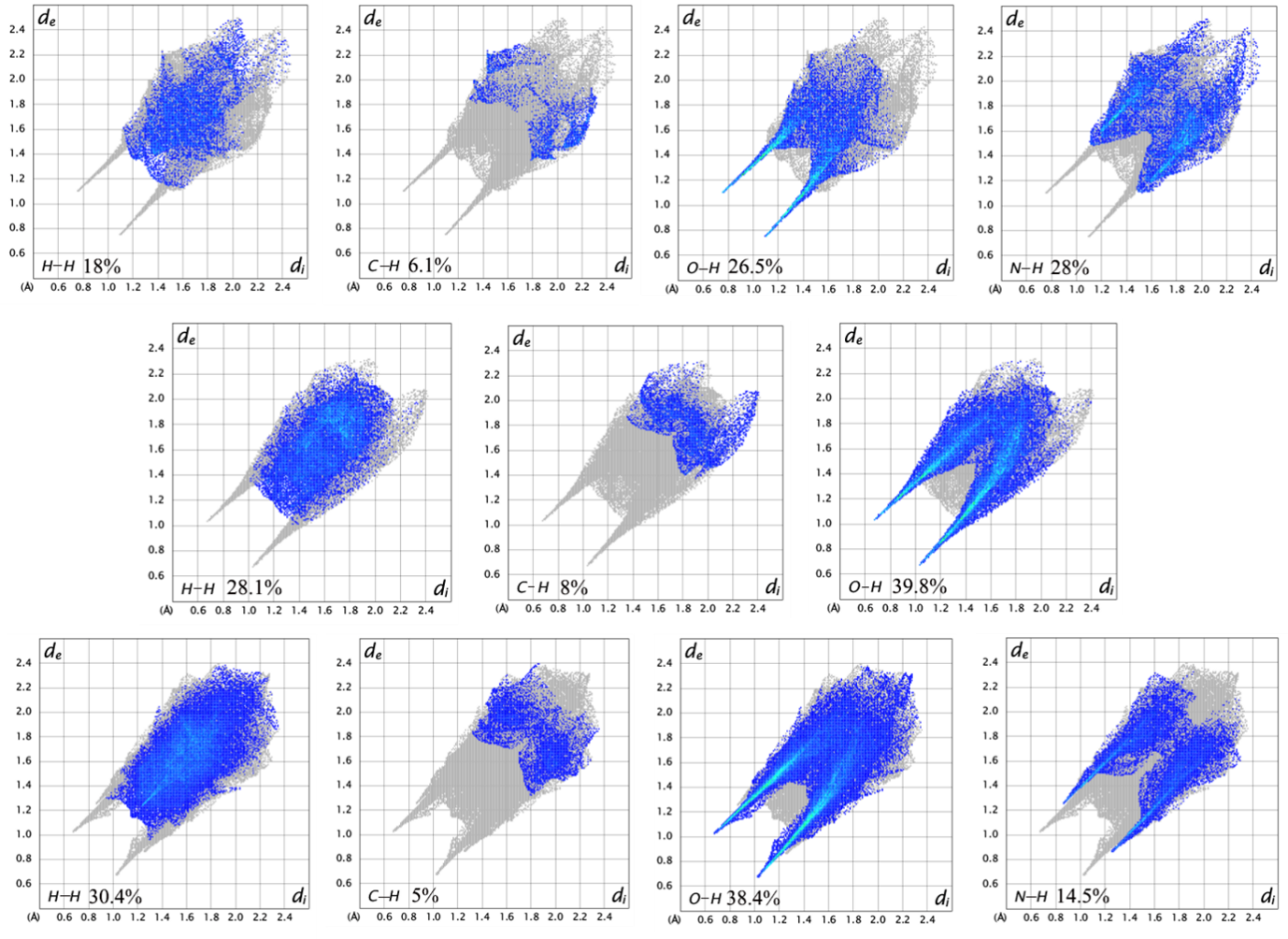


**(c)**

**(b)**

**(a)**

**Figure S3.** Hirshfeld Surface 2D fingerprint plots of (a) TMZ, (b) MYR∙H₂O, and (c) **2TMZ/MYR·4H₂O**.


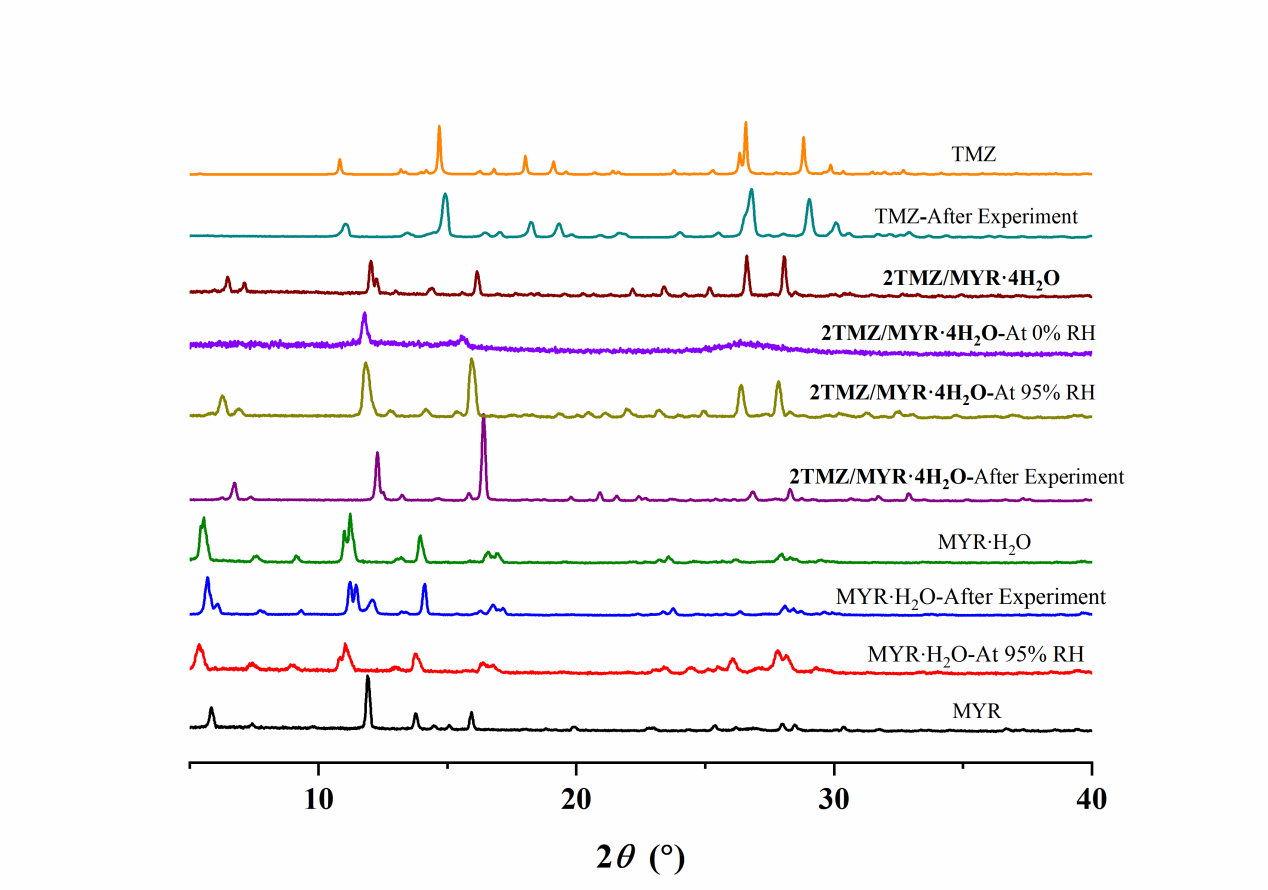


**Figure S4.** PXRD patterns of TMZ, MYR∙H₂O, **2TMZ/MYR·4H₂O** before and after DVS measurements.

**
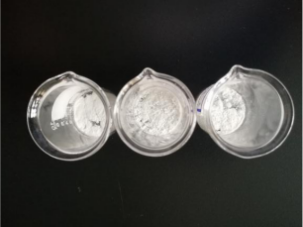

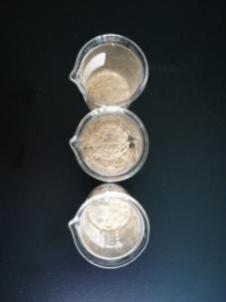

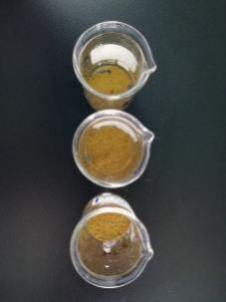
**
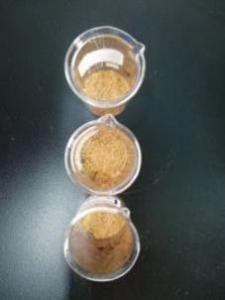


**(a)**

**(b)**

**
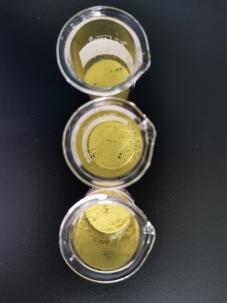

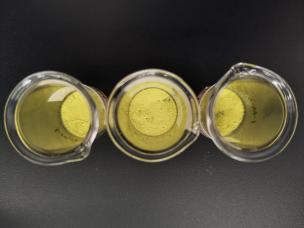

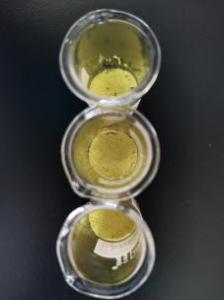
**
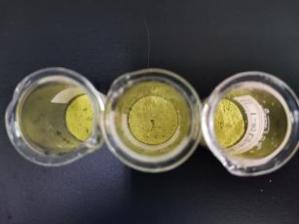


**Figure S5.** Photographs of (a) TMZ and (b) **2TMZ/MYR·4H₂O** under 40 °C/75% RH at 0, 1, 2, and 3 months.





**(a)**





**(b)**

**Figure S6.** PXRD patterns of (a) TMZ and (b) **2TMZ/MYR·4H₂O** under 40 °C/75% RH.


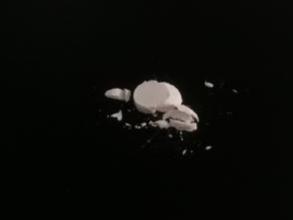

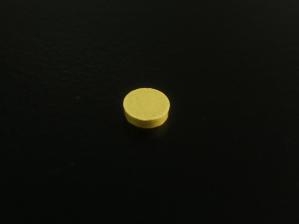

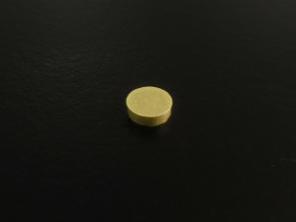


**(b)**

**(c)**

**(a)**

**Figure S7.** Photographs of (a) TMZ, (b) MYR∙H₂O and (c) **2TMZ/MYR·4H₂O** tablets under compaction pressure of 300 MPa.


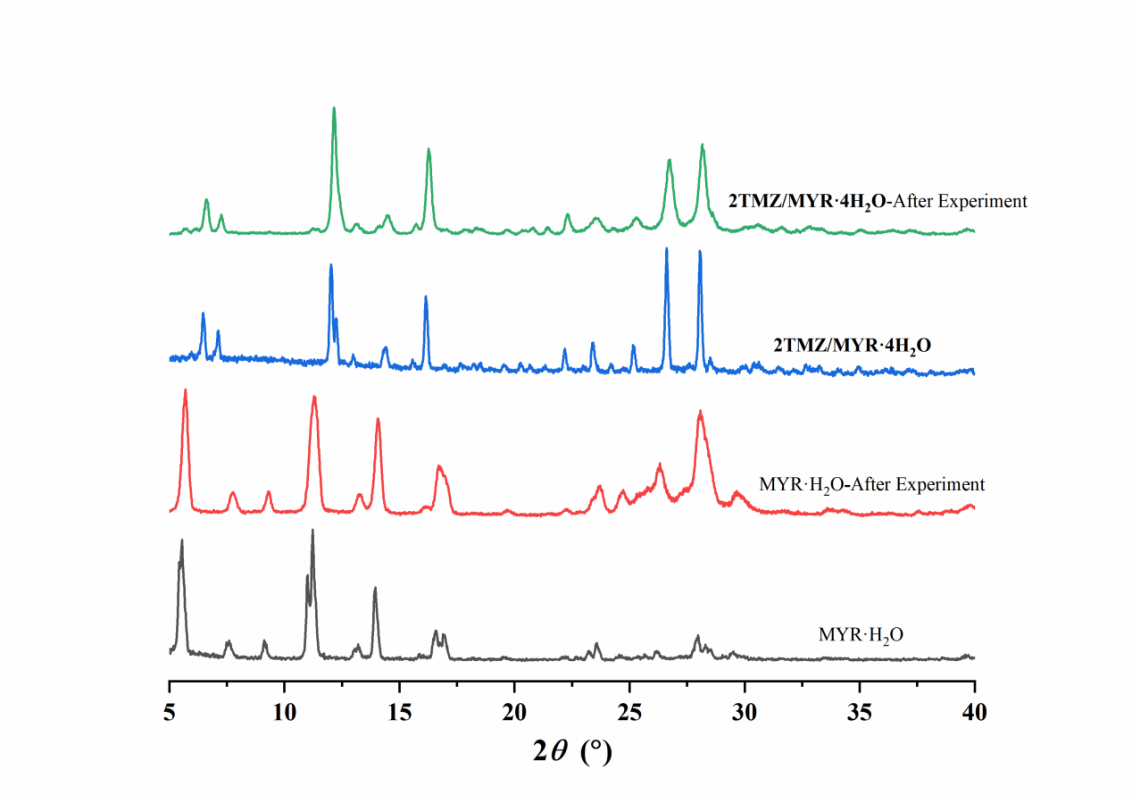


**Figure S8.** PXRD patterns of MYR∙H₂O and **2TMZ/MYR·4H₂O** before and after compaction experiments at 300 MPa.





**Figure S9.** PXRD patterns of TMZ, MYR∙H₂O, **2TMZ/MYR·4H₂O** before and after dissolution experiments.

**Table S1.** The hydrogen bonding distances and angles of **2TMZ/MYR·4H₂O**.

| hydrogen bond | H···A (Å) | D···A (Å) | ∠D‒H···A (°) | symmetry |
| --- | --- | --- | --- | --- |
| O2-H2⋅⋅⋅O3 | 1.96 | 2.686(2) | 146.6 | -x+1,-y+1,-z |
| O4-H4⋅⋅⋅O3 | 1.89 | 2.625(2) | 148.1 |  |
| O7-H7⋅⋅⋅O10 | 1.86 | 2.672(2) | 173.3 | -x+1,-y+2,-z+1 |
| O5-H5⋅⋅⋅O16 | 1.84 | 2.659(3) | 173.5 |  |
| O8-H8⋅⋅⋅O13 | 1.95 | 2.732(2) | 159.7 | x-1, y, z |
| N6-H6B⋅⋅⋅O11 | 2.57 | 3.208(2) | 131.9 | -x+1, -y+1, -z+1 |
| N6-H6B⋅⋅⋅O16 | 2.35 | 3.079(2) | 143.4 | -x+2, -y+1, -z+1 |
| N6-H6C⋅⋅⋅N7 | 2.30 | 3.104(3) | 155.0 | x+1, y, z |
| N12-H12A⋅⋅⋅O12 | 2.17 | 3.008(2) | 165.3 | -x, -y+1, -z |
| O15-H15B⋅⋅⋅O7 | 2.30 | 2.949(3) | 123.0 | x+1,y-1,z |
| O13-H13B⋅⋅⋅O9 | 2.11 | 2.974(3) | 155.0 |  |
